# miR156b-targeted *VvSBP8/13* functions downstream of the abscisic acid signal to regulate anthocyanins biosynthesis in grapevine fruit under drought

**DOI:** 10.1093/hr/uhad293

**Published:** 2024-01-02

**Authors:** Shuihuan Guo, Meng Zhang, Mingxin Feng, Guipeng Liu, Laurent Torregrosa, Xiaoqing Tao, Ruihua Ren, Yulin Fang, Zhenwen Zhang, Jiangfei Meng, Tengfei Xu

**Affiliations:** College of Enology, Northwest A&F University, Yangling, Shaanxi 712100, China; College of Food Science and Technology, Henan Agricultural University, Zhengzhou, Henan 450002, China; College of Enology, Northwest A&F University, Yangling, Shaanxi 712100, China; College of Enology, Northwest A&F University, Yangling, Shaanxi 712100, China; College of Enology, Northwest A&F University, Yangling, Shaanxi 712100, China; UMR LEPSE, Université de Montpellier , CIRAD, INRAE, Institut Agro, 34060 Montpellier, France; College of Enology, Northwest A&F University, Yangling, Shaanxi 712100, China; College of Enology, Northwest A&F University, Yangling, Shaanxi 712100, China; College of Enology, Northwest A&F University, Yangling, Shaanxi 712100, China; College of Enology, Northwest A&F University, Yangling, Shaanxi 712100, China; College of Enology, Northwest A&F University, Yangling, Shaanxi 712100, China; State Key Laboratory of Crop Stress Biology for Arid Areas, College of Horticulture, Northwest A&F University, Yangling, Shaanxi 712100, China

## Abstract

Anthocyanins are the primary color components of grapevine berries and wines. In cultivation practices, a moderate water deficit can promote anthocyanin accumulation in red grape skins. Our previous study showed that abscisic acid (ABA) plays a key role in this process. Herein, we identified a microRNA, vv-miR156b, that is generated in grapevine berries in response to drought stress, along with increasing anthocyanin content and biosynthetic structural gene transcripts. In contrast, vv-miR156b short tandem target mimic (STTM) function-loss callus exhibits the opposite phenotype. Results from *in vivo* and *in vitro* experiments revealed that the ABA-signaling-regulated transcription factor VvAREB2 binds directly to the ABA-responsive element (ABRE) of the *MIR156b* promoter and activates miR156b expression. Furthermore, two miR156b downstream targets, *VvSBP8* and *VvSBP13*, exhibited reduced grape anthocyanin content in their overexpressors but there was a contrary result in their CRISPR-edited lines, the decrease in anthocyanin content was rescued in miR156b and SBP8/13 double overexpressors. We further demonstrated that both *VvSBP8* and *VvSBP13*, encoding transcriptional repressors, displayed sufficient ability to interact with VvMYC1 and VvMYBA1, thereby interfering with MYB-bHLH-WD (MBW) repeat transcriptional complex formation, resulting in the repression of anthocyanin biosynthesis. Our findings demonstrate a direct functional relationship between ABA signaling and the miR156-SBP-MBW complex regulatory module in driving drought-induced anthocyanin accumulation in grape berries.

## Introduction

Grapevines are an important fruit crop worldwide. As an important trait and quality parameter, berry skin color determines market value and processing potential. Anthocyanins, a type of secondary metabolite, are the principal components of the red color of grapevine fruit skin and the resulting juice or wine. The content and composition of anthocyanins in grapes are primarily determined by genetic factors and depend on various environmental elements, including light, temperature, water, and nutrients [1, 2]. Water is one of the primary limiting factors during cultivation in most grape- and wine-producing regions. A moderate water deficit promotes anthocyanin accumulation in grapes during ripening [3]. However, the relevant molecular mechanism remains unclear, and the complicated regulatory network needs to be further elucidated.

In plants, anthocyanins are biosynthesized via the phenylpropanoid pathway, which involves a series of catalytic enzymes, including chalcone synthase, flavanone 3-hydroxylase 4-reductase (DFR), and uridine diphosphate glucose flavonoid 3-*O*-glucosyltransferase (UFGT). At the transcriptional level, the expression of these structural genes is directly regulated by the MYB-bHLH-WDR (MBW) protein complex, which comprises the R2R3-type MYB, basic helix–loop–helix (bHLH) and WD40 repeat (WDR). Currently, for grapevine, the MBW complex formation primarily include MYB TFs (such as VvMYBA1/2, VvMYBPA1/2/6/7, and VvMYB5a/5b), bHLH TFs (such as VvMYC1 and VvMYCA1), and WD40 proteins (such aVvWDR1 and VvWDR2) [4, 5]. Other TFs might also control anthocyanin accumulation by directly regulating the formation of MBW complexes [6]. Previous studies have established that COP1 (constitutively photomorphogenic1), SPL1/9 (SQUAMOSA promoter binding protein-like proteins 1/9), and JAZ (jasmonate ZIM-domain) participate in anthocyanin biosynthesis by altering the complexes [7–11].

MicroRNAs (miRNAs) are 20- to 24-nucleotide endogenous small RNAs that target mRNAs via imperfect sequence complementarity to silence or downregulate gene expression at the transcriptional and post-transcriptional stages [12, 13]. Increasing evidence supports miRNAs as crucial regulators of various processes, such as plant development and biotic and abiotic stress responses [14, 15]. miR156s, together with their downstream target *SPL* family of genes, are pivotal regulators of diverse biological processes, such as plant growth, fruit ripening, secondary metabolism, and stress response [16–22]. In *Arabidopsis*, miR156 targets *SPL9* and *SPL10* to directly activate miR172 and contributes to the regulation of anthocyanin production [23]. Recent evidence has revealed that miR156/*SPL* may play a role in the growth and ripening of grapevine berries [24]. miR156 coordinates the relationship between development and stress response in plants [10]. However, further investigation is necessary to determine how it responds to certain environmental cues from upstream and transfers the signals to downstream paths to perform this function.

**Figure 1:**
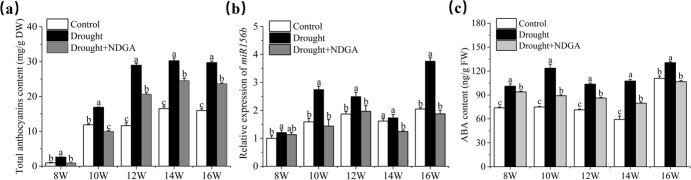
Total anthocyanins content (**a**), *vv-miR156b* expression pattern (**b**), and ABA content (**c**) under control and drought conditions during grape growth. The *X*-axis (8–16W) indicates weeks after flowering. NDGA is an inhibitor of ABA synthesis.

Several studies have reported that miRNAs associated with an abscisic acid (ABA)-independent signal transduction pathway CBF/DREB (*c*-repeat-binding factor/drought response element-binding) TF increase tolerance to drought stress in tomatoes and wheat [25, 26]. For example, miR156 responds to increased endogenous ABA levels induced by drought stress [16, 27, 28]. Li *et al.* [29] speculated that anthocyanin synthesis is highly related to endogenous ABA levels and drought-responsive genes, such as the dehydration-responsive element binding (DREB) protein, ABA-responsive element (ABRE)-binding protein, NAM, ATAF, and CUC regulators. These drought-responsive genes play a significant role in controlling numerous genes that respond to drought [27, 28, 30], indicating the necessity to investigate a complicated network between ABRE and DRE *cis*-elements. In addition, further studies should be investigated whether *cis*-element involvement in complex overlapping networks links ABA signaling and miR156 in the pathway of drought-promoted anthocyanin synthesis in grapes.

Our previous study showed that ABA signaling is more crucial than other phytohormones for drought-induced anthocyanin formation in grape berries [31]. Therefore, we performed small RNA sequencing (RNA-seq) and identified an miRNA, miR156b, that was induced by drought treatment in grapevine berries. A significant positive correlation was observed between vv-miR156b expression, ABA levels, and anthocyanin accumulation. We then systematically characterized the function of vv-miR156b and showed that the VvAREB2 protein could directly bind to the ABRE in the *vv-MIR156b* promoter and activate its expression. This resulted in the repression of two downstream targets (*VvSBP8* and *VvSBP13*), thereby weakening their function as competitors and disturbing MBW complex formation by interacting with the two TFs VvMYBA1 and VvMYC1. Our results provide a direct functional regulatory module of the ABA signaling-VvAREB2-miR156b-*VvSBP8/13-*MBW complex pathway that mediates anthocyanin accumulation in response to drought stress.

## Results

### miR156b correlates with anthocyanins and abscisic acid contents in grapes under drought 

Our previous data showed that the expression level of vv-miR156b in grape berries was significantly upregulated under water deficit conditions, using small RNA-seq. To further investigate the regulatory pattern between the expression level of vv-miR156b and anthocyanin accumulation under drought stress, the grapevines were subjected to drought treatments starting 4 weeks after flowering (WAF) until the full grape ripening stage. The results showed that the anthocyanins content in grape berry skins rapidly increased from 10 WAF, and drought treatment effectively promoted its accumulation compared with the control (Fig. 1a). Moreover, we examined mature vv-miR156b expression, which increased at 10 WAF under drought treatment (Fig. 1b), suggesting that the expression pattern of vv-miR156b is positively correlated with anthocyanin content. Our previous study showed that ABA signaling plays a key role in drought-driven anthocyanin accumulation in grape berries compared with other phytohormones [31]. In this study, the ABA content was upregulated in response to drought (Fig. 1c), but the miR156b expression and anthocyanins accumulation decreased when these grape berries were treated with nordihydroguaiaretic acid (NDGA) (Fig. 1a and b), which is an ABA inhibitor that strongly limits ABA biosynthesis. In addition, ABA content and drought effects also reduced following NDGA treatment (Fig. 1c). This result indicates that drought stress promotes anthocyanin accumulation, which correlates with miR156b expression, accompanied by a strong link with ABA signaling in grapevine berries.

### miR156b enhances drought-induced anthocyanin accumulation in grapevine and *Arabidopsis*

**Figure 2:**
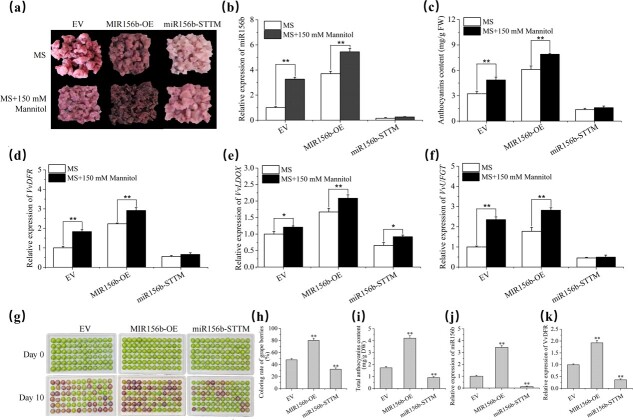
Regulation of anthocyanin biosynthesis by vv-miR156b. **a** Phenotype of grape calli grown in MS (control) or MS + 150 mM mannitol (simulated drought) medium. MIR156b-OE, vv-MIR156b overexpression; miR156b-STTM, vv-miR156b mutant created using short tandem target mimic (STTM) technology. **b** Expression levels of mature vv-miR156b in EV and transgenic grape calli determined using stem-loop qRT–PCR (5.8S rRNA and U6 were used as the internal controls). **c** Contents of anthocyanins in EV and transgenic grape calli. **d**–**f** Expression levels of anthocyanin biosynthesis structural genes *VvDRF*, *VvLDOX*, and *VvUFGT* determined using qRT–PCR (*VvACTIN* and *VvUBI* were used as internal references). **g**–**k** Anthocyanin accumulation phenotypes (**g**), coloring rate (**h**), anthocyanin content (**i**), and relative expressions of vv-miR156 (**j**) and *VvDFR* (**k**) in transient transformed grape berries.

To explore the role of vv-miR156b in grape anthocyanin accumulation, we obtained several stable transgenic grape calli overexpressing (OE) the vv-miR156b precursor under the 35S promoter to achieve vv-miR156b function (MIR156b-OE) . The miR156b-STTM (STTM, short tandem target mimic) sequence was overexpressed in grape calli driven by the 35S promoter to suppress the expression of vv-miR156b (miR156b-STTM) (Supplementary Data Fig. S1a). The calli were cultured on a medium supplemented with kanamycin until resistant and stable transgenic calli were generated (Supplementary Data Fig. 2a). In *MIR156b-OE* lines, the expression level of mature vv-miR156b detected using stem-loop qRT–PCR was significantly downregulated relative to that in the miR156b-STTM and empty vector (EV) lines (Fig. 2b). Consistently, the anthocyanin content in the *MIR156b-OE* calli increased while miR156b-STTM decreased (Fig. 2c). In addition, the transcript levels of late anthocyanin biosynthetic structural genes, such as *VvDFR*, *VvLDOX*, and *VvUFGT*, were measured in these transgenic calli and found to be upregulated in the *MIR156b-OE* calli compared with the control. In contrast, the miR156b-STTM lines were significantly lower than those in the EV lines (Fig. 2d–f). These results demonstrate that vv-miR156b increased anthocyanin biosynthesis and induced the expression of anthocyanin biosynthetic genes in grape cell derivatives. Additionally, vv-miR156b overexpression and suppression plasmids were transiently transformed in the grape berries. Consistent with the results in grape calli, the overexpression of vv-miR156b promoted the accumulation of anthocyanins in grape berries, whereas its suppression inhibited anthocyanin accumulation (Fig. 2g–k). These results further support the positive role of miR156b in mediating anthocyanin accumulation under drought stress.

**Figure 3:**
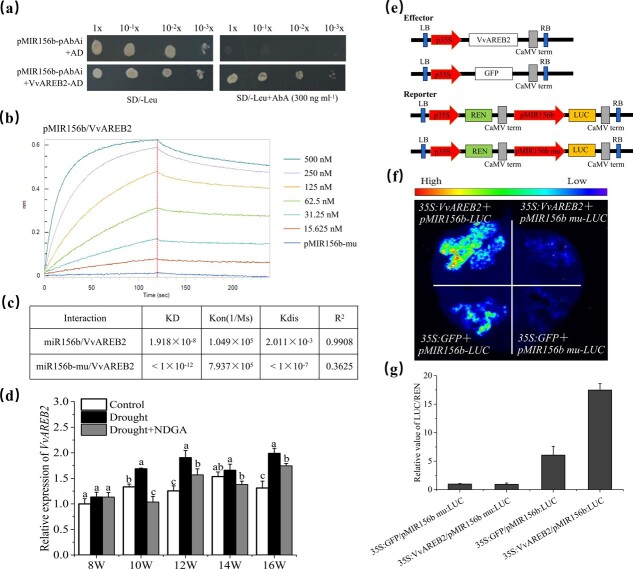
VvAREB2 directly binds to the *vv-MIR156b* promoter and activates its expression. **a** Y1H assay showing the direct binding of VvAREB2 to the *MIR156b* promoter (pMIR156b). **b** BLI assay showing the association and dissociation curves of *MIR156b* or *MIR156b-mu* promoter (*pMIR156b-mu*) with VvAREB2 protein. Immobilized on streptavidin biosensor tips, the biotinylated *MIR156b* promoter was incubated over a range of quantities of soluble VvAREB2 protein (15.625–500 nM). Biotinylated *MIR156b* promoter mutant was used as a control. **c** Analysis of kinetic rate constants and affinities between *MIR156b* or *MIR156b-mu* with the VvAREB2 protein in the BLI assay. BLI assay of the *MIR156b* promoter mutant with various VvAREB2 protein concentrations is shown in Supplementary Data Fig. S3. **d** Expression levels of VvAREB2 during grape growth under control and drought stress. The *X*-axis (8–16W) indicates weeks after flowering. NDGA is an inhibitor of ABA synthesis. **e** Effector and reporter constructs used in the tobacco transient expression assay are shown schematically. (**f**) Dual-LUC activity assay shows that VvAREB2 activates the expression of *MIR156b*. **g** Quantitative analysis of relative LUC to REN activity. Expression of REN was used as an internal control. Values given are means of biological triplicates (± standard deviation).

To evaluate the role of miR156b in drought-induced anthocyanin biosynthesis, EV and miR156b transgenic lines were transferred to a medium containing 150 mM mannitol to simulate drought. A significant increase in anthocyanin content, deeper callus color, and higher miR156b expression levels were observed in *MIR156b-OE* grape calli lines compared with EV calli, whereas values for miR156b-STTM did not change (Fig. 2a–c). In addition, the expression levels of *VvUFGT*, *VvLDOX*, and *VvDFR* showed a similar pattern to the phenotype in these transgenic calli upon drought treatment (Fig. 2d–f). Similarly, we overexpressed vv-MIR156b, driven by the *Arabidopsis* drought-inducible *AtRD29A* promoter in heterologous *Arabidopsis thaliana* plants, to facilitate its function across species barriers. *RD29A:MIR156b*-overexpressing lines produced stem-rosette nodes with red coloration (Supplementary Data Fig. S2a). The *MIR156-OE* lines were confirmed by semi-qRT–PCR analysis of mature vv-miR156b (Supplementary Data Fig. S2b), and a significant increase in anthocyanin content was detected (Supplementary Data Fig. S2c). Therefore, we demonstrated that miR156b promotes the accumulation of anthocyanins in grape under drought conditions.

**Figure 4:**
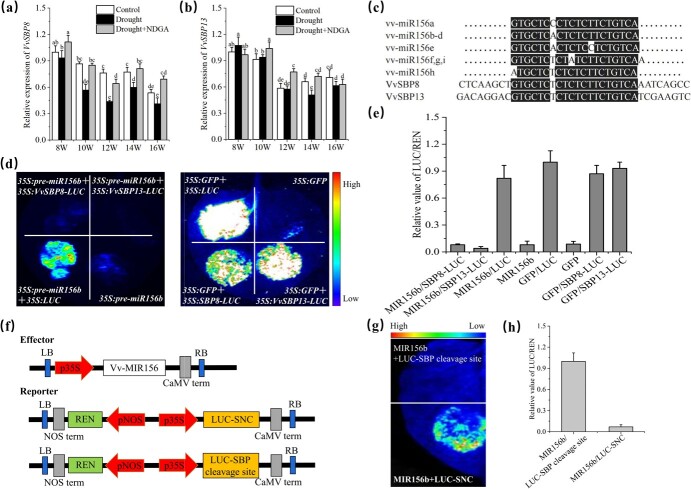
vv-miR156b targets and regulates the expression of *VvSBP8/13*. **a**, **b** Quantitative assessment of *VvSBP8* and *VvSBP13* transcript levels during grape growth under control and drought conditions. Data are means ± standard deviation from three separate events. **c** Alignment of mature vv-miR156s with *VvSBP8/13* target sites. **d** LUC dual test confirming vv-miR156b suppression of *VvSBP8/13*. The *VvSBP8/13-LUC* gene was driven by the 35S promoter. Reporters were employed to transiently co-transform tobacco leaves, along with the *35S:pre-miR156b e*ffector. 35S-driving GFP was used as effector control and 35S driving was used as reporter control. **e** LUC/REN ratio of chemiluminescence. Data are means ± standard deviation from three separate events. **f** Schematic representation of the effector and reporter constructs. **g**, **h** Level of suppression of *VvSBP8/13* targets caused by vv-miR156 was measured using a dual-LUC reporter assay. *35S:vv-MIR156b* (miR156b precursor) was co-transformed with *VvSBP8/13* cleavage sites fused to the ORF of the LUC gene. Synonymous negative control (SNC) is negative control with the disruption of *VvSBP8/13* cleavage sites while maintaining the same amino acid sequence.

### VvAREB2 directly binds to the miR156b promoter to activate its expression

Because ABA content was positively correlated with miR156b abundance under drought stress, we speculated that ABA signaling-related genes may directly regulate miR156b expression. To test this hypothesis, we cloned the *vv-MIR156b* promoter fragment into a pAbAi plasmid and performed a yeast one-hybrid (Y1H) screening assay using the grape skin cDNA library to identify the transcriptional factors implicated in the upstream pathway of vv-miR156b. We obtained the candidate protein VvAREB2, an ABA-responsive TF that requires ABA for full activation, and performed experiments to confirm the binding of VvAREB2 to the *vv-MIR156b* promoter. The Y1H assay confirmed that VvAREB2 binds to the *vv-MIR156b* promoter (Fig. 3a). Further analysis revealed the presence of ABRE *cis*-elements in the *MIR156b* promoter (Supplementary Data Fig. S3). A bio-layer interferometry (BLI) assay using a specific ABRE *cis*-element-containing probe immobilized on streptavidin biosensor tips showed that purified VvAREB2 protein bound explicitly to the ABRE *cis*-elements in the *vv-MIR156b* promoter, whereas the mutated ABRE *cis*-element in the promoter blocked the binding with VvAREB2, thus indicating the specificity of VvAREB2 protein binding to ABRE-acting elements in the *vv-MIR156b* promoter (Fig. 3b). To further quantify the binding affinities, we compared the kinetic values of the VvAREB2 protein to the *MIR156b* promoter (*pMIR156b*) and *MIR156b* promoter with the ABRE *cis*-element mutant (*pMIR156b-mu*). The *K*_D_ value of the VvAREB2-*pMIR156b* binding was significantly higher than that of VvAREB2-*pMIR156b-mu* binding (Fig. 3c; Supplementary Data Fig. S4), indicating that VvAREB2-*pMIR156b* has extremely strong affinity for the ABRE *cis*-element.

Next, we performed a transient expression study to determine whether VvAREB2 directly regulates the expression of vv-miR156b. *Agrobacterium* strains carrying *p35S:VvAREB2* effector and *pMIR156b:LUC* reporter were injected into tobacco leaf epidermal cells. Following incubation in darkness at 25°C for 2–3 days, the relative LUC to REN activity was examined. Co-expression of the VvAREB2 protein stimulated luciferase (*LUC*) reporter gene activities driven by the endogenous promoters of *vv-MIR156b.* However, this activation was eliminated after mutating AREB element in the promoter, GFP empty vector was used as the negative control (Fig. 3e and f). Relative LUC activity normalized to REN activity is shown in Fig. 3g. The *VvAREB2* transcripts were higher under drought stress but significantly decreased under drought with the ABA inhibitor NDGA (Fig. 3d). Thus, we concluded that drought could increase *VvAREB2* levels via ABA signaling, then VvAREB2 protein directly binds to the promoter of *vv-MIR156b* via ABRE-acting element, and activates its expression.

### V‌V-miR156b targets and represses *VvSBP8/13* expression

In plants, miR156 typically targets the genes belonging to the SBP family. To confirm whether a specific SBP member acts downstream of vv-miR156b under drought stress, we previously analyzed all SBP family member transcripts and found that *VvSBP8* and *VvSBP13* exhibited opposite expression trends to vv-miR156b under drought stress (Fig. 4a and b; Supplementary Data Figs S5 and S1b). We examined the cleavage sites of the VvSBP genes and mature vv-miR156s sequences to confirm the regulation of VvSBP8 and 13 transcripts by vv-miR156b (Fig. 4c). We further demonstrated miR156–target interactions *in vivo* using a transient assay conducted in tobacco leaves. When *pre-miR156b* was co-expressed with the *VvSBP8/13-LUC* reporter, as opposed to the empty LUC reporter (positive control), the LUC signal intensity was significantly reduced. Moreover, no LUC signal intensity from the *pre-miR156b* or GFP EV was observed, whereas co-expression of the GFP EV and *VvSBP8/13-LUC* reporter resulted in an LUC signal because it had no capacity for targeting (negative control) (Fig. 4d and e). These results indicated that vv-miR156b could target both *VvSBP8* and *VvSBP13* and suppress their expression.

Moreover, the open reading frame (ORF) of *VvSBP8/13* contains putative vv-miR156b cleavage sites. Co-expression of the miR156b precursor (*35S:MIR156b*) with *VvSBP8/13* cleavage sites fused to the ORF of the firefly LUC gene resulted in reduced LUC/REN activity, which was significantly lower than that observed in the synonymous negative control (SNC), which is the negative control with the disruption of *VvSBP8/13* cleavage sites while maintaining the same amino acid sequence (Fig. 4f–h). These findings suggest that vv-miR156b targets the *VvSBP8* and *VvSBP13* cleavage sites in grapes. Correspondingly, the expression level of *VvSBP8/13* decreased under drought stress and recovered when treated with an ABA inhibitor (Fig. 4a and b). We suggest that drought-induced ABA signaling can increase vv-miR156b capacity, thereby recognizing *VvSBP8/13* while targeting its cleavage sites and suppressing its expression.

### Vv-miR156b inhibits the function of *VvSBP8/13* in reducing anthocyanin accumulation

**Figure 5:**
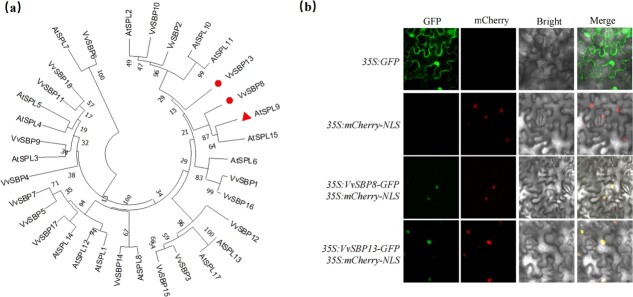
Phylogenetic analysis of SPL/SBP amino acids from *V. vinifera* and *Arabidopsis* (**a**) and the subcellular location of VvSBP8 and VvSBP13 (**b**).

Phylogenetic analysis of *Vitis* VvSBP and *Arabidopsis* AtSPL amino acid sequences revealed that VvSBP8/13 is highly homologous to AtSPL9 (Fig. 5a), which belongs to a recognized anthocyanin-specific branch in other plant species [8]. This prompted us to study the role of VvSBP8/13 in regulating anthocyanin accumulation in grapevine berries. First, we determined the subcellular location of the VvSBP8 and VvSBP13 TFs, and *p35S:VvSBP8-GFP* and *p35S:VvSBP13-GFP* vectors were individually introduced into tobacco plants to observe the GFP signal. The *p35S:GFP* and mCherry-NLS nuclear localization marker protein were used as negative and positive controls, respectively. We used confocal microscopy to observe the VvSBP8-GFP and VvSBP13-GFP fusion protein signals, and both fluorescence signals were clearly visible in the nucleus, indicating that VvSBP8 and VvSBP13 were localized to the nucleus (Fig. 5b).

**Figure 6:**
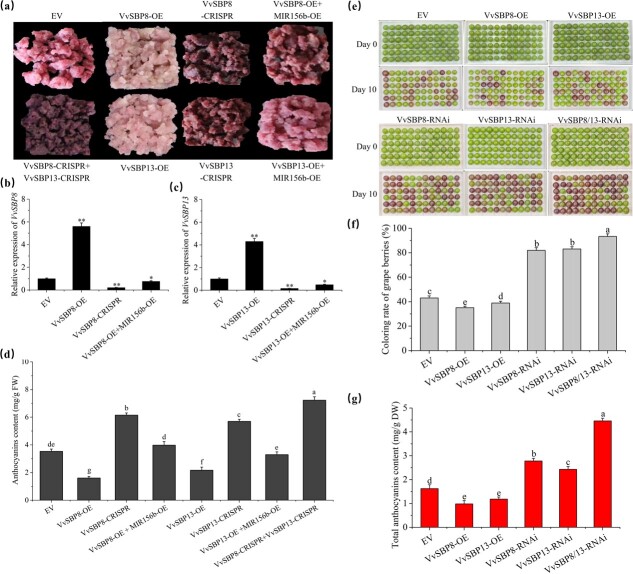
*VvSBP8* and *13* negatively affect anthocyanin accumulation and are repressed by miR156b. **a** Functional characterization of transgenic grape calli. **b**, **c** Relative expression levels of *VvSBP13* and *VvSBP8* in transgenic calli. *VvACTIN* and *VvUBI* were used as endogenous controls. **d** Contents of anthocyanins in EV and transgenic calli. **e**–**g** Anthocyanin accumulation phenotypes (**e**), coloring rate (**f**), and anthocyanin contents (**g**) in transient transformed grape berries.

To evaluate the function of *VvSBP8/13*, we overexpressed and CRISPR-genome-edited *VvSBP8* and *VvSBP13* in red grape callus to obtain *VvSBP8-OE*, *VvSBP13-OE, VvSBP8-CRISPR*, and *VvSBP13-CRISPR*. In addition, *VvSBP8-CRISPR + VvSBP13-CRISPR* double callus lines were further generated in case of possible redundant function (Fig. 6a). Gene expression levels were confirmed using qRT–PCR (Fig. 6b and c), and protein levels in *VvSBP8/13-OE* were detected using anti-GFP detection (Supplementary Data Fig. S6). The results showed that the overexpression of *VvSBP8* and *VvSBP13* individually reduced the anthocyanin content; on the contrary, the CRISPR-edited *VvSBP8* and *VvSBP13* caused an increase in anthocyanin content, when *VvSBP8* and *VvSBP13* were simultaneously edited, showing a greater increase in anthocyanin content (Fig. 6a and d). We also generated *VvSBP8-OE* and *VvSBP13-OE Arabidopsis* stable lines (Supplementary Data Fig. S7a) and confirmed the presence of immunoreactive bands by using anti-GFP (Supplementary Data Fig. S7b). The anthocyanin contents in *VvSBP8-OE* and *VvSBP13-OE* were much less than in the wild-type (WT) and EV transgenic lines (Supplementary Data Fig. S7c), highlighting their negative role in regulating anthocyanin content. Moreover, the overexpression and RNA interference vectors for *VvSBP8* and *VvSBP13* were transiently transformed in grape berries. Consistent with the results for grape calli and *Arabidopsis* lines, the overexpression of VvSBP8/13 in grape berries inhibited anthocyanin biosynthesis, whereas the RNAi suppression contributed to anthocyanin accumulation (Fig. 6e–g). These results suggest that *VvSBP8* and *VvSBP1*3 are functionally redundant in inhibiting anthocyanin accumulation.

To confirm that vv-miR156b is involved in this process, we overexpressed *vv-MIR156b* in the *VvSBP8-OE* or *VvSBP1*3-*OE* transgenic callus background and verified mature vv-miR156b expression (Supplementary Data Fig. S8). Anthocyanins were reduced in both *VvSBP8-OE + MIR156b-OE* and *VvSBP13-OE + MIR156b-OE* transgenic calli (Fig. 6a and d). The considerably lower expression levels of *VvSBP8* and *VvSBP13* displayed in the *VvSBP8-OE+MIR156b-OE* and *VvSBP13-OE+MIR156b-OE* co-transgenic callus lines were similar to those in the EV transgenic lines, which was antagonistic to the upregulation in *VvSBP8-OE* and *VvSBP13-OE* individual lines (Fig. 6b and c)*.* In summary, the gain of vv-miR156b in grape calli resulted in the downregulation of *VvSBP8/13* target genes, which suppressed grape anthocyanin biosynthesis.

### VvSBP8/13 displaces VvMYC1/VvMYBA1 from the MBW complex

**Figure 7:**
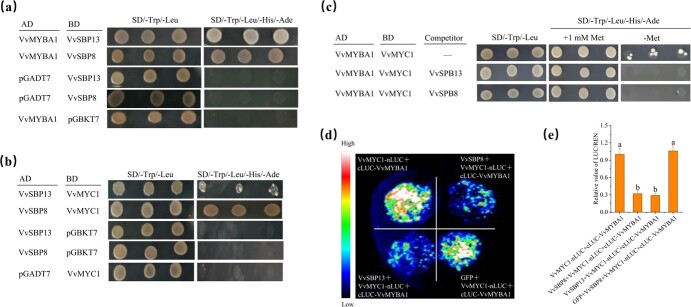
VvSBP8/13 affects the interaction of VvMYC1-VvMYBA1. **a**, **b** Y2H growth assay. Specified AD and pBridge BD fusion proteins were introduced into AH109 yeast cells and the cells were then cultured on SD LT or SD LTHA plates. **c** Assay for Y3H competition. Plasmids encoding AD-VvMYBA1 and pBridge-VvMYC1, as well as potential rivals VvSBP8/13, were transformed into AH109 cells. As a transformation control, SD LT plates were employed. **d** Split-LUC assays show VvSBP8/13 inhibition of MBW complex proteins VvMYC1 and VvMYBA1 in tobacco leaves. VvSBP8, VvSBP13, and GFP (negative control) were rapidly co-expressed together with VvMYC1-nLUC and cLUC-VvMYBA1. **e** Luminescence intensities of different combinations of three biological replicates were quantified.

In grapevines, the MBW complex, which comprises R2R3-MYB, bHLH, and WD40 proteins, influences the expression of anthocyanin biosynthesis genes [32, 33]. The MYB TFs VvMYBA1 and VvMYBA2 and bHLH TF VvMYC1 were identified as components of the MBW complex [6]. Here, the yeast two-hybrid (Y2H) assay showed that both VvSBP8 and VvSBP13 could interact with VvMYBA1 and VvMYC1 (Fig. 7a and b). Therefore, we used the yeast three-hybrid (Y3H) assay to test whether they compete for binding to either VvMYBA1 or VvMYC1. VvMYBA1 was fused to the Gal4 activation domain (AD-VvMYBA1), and the pBridge vector was used as a BD vector to express VvMYC1 and VvSBP8/13, where VvMYC1 is driven by the constitutive yeast promoter *ADH1* and VvSBP8/13 is expressed under the *Met25* promoter. We co-expressed AD-VvMYBA1 with either pBridge-VvMYC1-VvSBP8 or pBridge-VvMYC1 in AH109 yeast cells. Because *VvSBP8/13* expression driven by the *Met25* promoter is repressed in media containing 1 mM methionine, whereas it is expressed in media lacking methionine, the co-expressed yeast cells were grown on the SD−Trp−Leu−His−Ade medium containing 1 mM methionine for transformation growth and selective SD−Trp−Leu−His−Ade−Met medium for examining competition. The results demonstrated that the negative control, pBridge-VvMYC1, which lacks the competitor *VvSBP8/13*, could still grow and had no effect on the interaction between VvMYBA1 and VvMYC1. However, VvSBP8/13 expressed under methionine-negative conditions resulted in little growth (Fig. 7c), indicating that VvSBP8/13 can displace VvMYBA1 and VvMYC1.

The split-LUC assay was then repeated using tobacco leaves expressing VvMYC1-nLUC and cLUC-VvMYBA1 in addition to VvSBP8-GFP, VvSBP13-GFP, or GFP control. In tobacco leaves co-expressing VvMYC1-nLUC and cLUC-VvMYBA1 with VvSBP8-GFP or VvSBP13-GFP, compared with the GFP control vector, significantly reduced luciferase activity was observed (Fig. 7d). The results of the relative LUC/REN activity assay were similar to those of the phenotypic observations (Fig. 7e). Therefore, VvSBP8 and VvSBP13 interrupt VvMYC1 and VvMYBA1 binding to form the BMW complex and inhibit MBW complex formation, resulting in decreased anthocyanin biosynthesis and accumulation in grapes.

## Discussion

**Figure 8:**
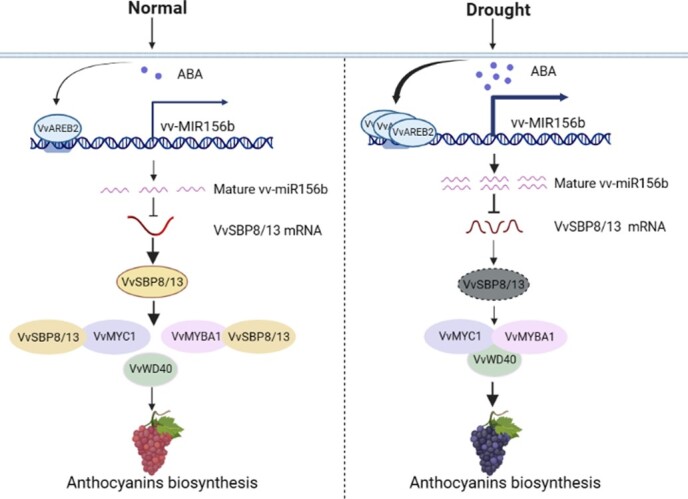
Model describing how drought mediates ABA-induced anthocyanin biosynthesis via the VvAREB2-miR156b-VvSBP8/13 pathway in grape berries. In this model, drought induces an increase in ABA levels, which promotes VvAREB2 to bind and activate the expression of the vv-miR156b promoter, thereby targeting the inhibition of VvSBP8/13. This attenuates the disruptive effect of VvSBP8/13 on the formation of the VvMYC1-VvMYBA1-VvWD40 (MBW) complex and ultimately promotes the accumulation of anthocyanins in grape berries under drought.

Numerous plant species and organs have been studied to determine how anthocyanins accumulate under drought stress [34]. However, the exact mechanism through which drought stress induces anthocyanin production remains unclear. According to certain authors, increasing ABA levels may be the cause of the elevated anthocyanin content observed in drought-stressed plants [35–37]. However, it is unclear how miR156s function or whether other endogenous components are also involved in this process. The pathway of miR156/SBP modules from upstream drought stress to downstream anthocyanin formation in grapevine fruit has not yet been studied. Here, we identified that the VvAREB2 TF can respond to an ABA-dependent signal transduction pathway upon drought stress, bind directly to the AREB elements on the promoter of *vv-MIR156b*, and increase the expression of miR156b. This results in the decrease of its key downstream target genes *VvSBP8* and *VvSVP13*, thereby inhibiting its role as a competitor to displace the VvMYBA1 or VvMYC1 protein from the MBW complex, resulting in the intensive MBW complex formation and activities that are required for anthocyanin accumulation (Fig. 8).

### Mechanism of miR156b response to drought stress

Non-coding RNAs are not translated into proteins and are divided into small (miRNAs) and long non-coding RNAs (lncRNAs) based on their length [38, 39]. Recently, increasing evidence shows that miRNAs repress mRNA expression and are important regulators of a range of biological processes in plants [40–42]. Numerous miRNAs can be expressed in response to drought stress [3, 43]; however, the mechanisms and regulatory networks involved in this process have not been completely explored. According to Boopathi [16], miRNA156 is expressed in response to increased endogenous ABA levels [3]. In our study, we provided genetic and molecular evidence that the ABA-responsive TF VvAREB2 could be induced by drought stress and positively correlated with ABA levels, which are major transcriptional regulators in the ABA-mediated stress signal transduction pathway [44, 45]. Among them, AREB2 is a major TF implicated in drought stress resistance, and water stress can promote the expression of AREB2 in *Arabidopsis* for ABA signaling [46]. We provide genetic and molecular evidence that the ABA-responsive TF VvAREB2 directly binds to the promoter of the miR156b precursor and activates its expression. VvAREB2 is a key player in ABA-induced vv-miR156b expression under drought stress. Designated ABREs can enhance drought stress tolerance in plants [30]. We also verified the specific ABRE elements in the miR156b promoter that bound with VvAREB2 but not with the mutant probe, suggesting a model in which drought elevated the miR156b expressions. Therefore, this conclusion might offer a rationale for the correlation. More research is needed to identify whether alterations in miR156b are an indirect result of their influence on drought-induced anthocyanin accumulation or whether they directly participate in this process.

### Function of miR156b-regulated *VvSBP* genes in anthocyanin accumulation under drought

Recently, growing evidence suggests that miRNAs are crucial for anthocyanin synthesis by repressing mRNA expression [40–42], and the expression of numerous particular miRNAs can be induced by drought stress in plants [3, 43]. The overexpression of vv-miR156b significantly increased anthocyanin accumulation in transgenic grape calli and in *Arabidopsis*, and this effect was enhanced by drought treatment, but not in CRISPR-edited calli mutant lines. miR156s target SPL/SBP to regulate plant development, fruit ripening, secondary metabolism, and abiotic stress responses [8, 24, 47]. Therefore, determining whether miR156-regulated SBP genes participate in the modulation of anthocyanin levels is necessary. We provide evidence that vv-miR156b targets and represses the expressions of *VvSBP8* and *VvSBP13*. The *MIR156-OE* callus phenotype in these experiments was nearly identical to that of the *VvSBP8* and *VvSBP13* CRISPR-edited mutants, indicating that SBP activity was completely or almost completely eliminated by the *35S:MIR156b* transgenic callus. In addition, vv-miR156b targeted the ORF of *VvSBP8/13*, thereby suppressing its expression. Therefore, this miRNA may provide new targets for the bioengineering of anthocyanins in grapevines. We also discovered an additional role of the miR156b–SBP model in modulating metabolic flux in the flavonoid biosynthetic pathway in grapes.

Anthocyanins are secondary metabolites of flavonoids biosynthesized via the phenylalanine pathway [48, 49]. Their biosynthesis is co-regulated by TFs and structural genes [50]. In the present study, *VvUFGT*, *VvLDOX*, and *VvDFR*, three genes involved in the regulatory pathway of anthocyanin biosynthesis, were detected in miR156b and VvSBP8/13 overexpressing and CRISPR-edited individual or simultaneous transgenic lines, and these structural genes were misregulated, where drought treatment promoted this effect. The expression of anthocyanin biosynthesis genes was suppressed by elevated levels of VvSBP8/13.

### Function and mechanism of VvSBP8/13 genes in regulating anthocyanin accumulation in grapes

The regulated activity of SPLs/SBPs is essential for cell growth [51, 52], embryo development [53], trichome initiation [54], and fertility [55]. Previous studies have reported that SPL proteins can modulate metabolic flux in the flavonoid biosynthetic pathway [8, 21]. Here, we report that the overexpression of *VvSBP8/13* reduces anthocyanin accumulation, in agreement with the results obtained for the ortholog SPL9 in *Arabidopsis* [8]. We demonstrated that *VvSBP8* and *VvSBP13* were repressed by miR156b, and the drought-induced miR156b caused a decrease in their expression, suggesting that SBP genes are involved in anthocyanin regulation, particularly by abiotic factors.

In plants, anthocyanin biosynthetic genes are modulated by a ternary MBW complex or independent MYB TFs [22, 56, 57]. MYB and bHLH TFs are critical for regulating anthocyanin biosynthesis in higher plants and have been detected in several species, such as *Arabidopsis* [58], apple [59, 60], and grapevine [61]. Previous studies have demonstrated that, in *Arabidopsis*, increased miR156 activity promotes anthocyanin accumulation by targeting SPL9, which competes with the bHLH TF TT8 to bind the MYB TF PAP1, thereby preventing the formation of the MYB-bHLH-WD40 (MBW) protein complex [8]. PpSPL10 and PpSPL13 in Chinese sand pears interact with PpMYB10 [19], and PpSPL1 in blood-fleshed peaches inhibits the expression of PpMYB10.1, possibly by inhibiting the assembly of the MBW protein complex [20]. Blueberry VcSPL12 interacts with VcMYBPA1 to coordinate the accumulation of chlorophyll and anthocyanins [22]. Here, a series of approaches demonstrated that either VvSBP8 or VvSBP13 can displace VvMYC1 and VvMYBA1 individually and disrupt their binding, indicating that the functions of the SPL family are well conserved in plants.

## Conclusion

In summary, we constructed a model in which the drought-ABA signal-VvAREB2-miR156b-VvSBP8/13-MBW (VvMYC1-VvMYBA1-VvWD40) module regulates anthocyanin accumulation in grapevine berries under water-deficit conditions. In the present study, we focused on the role of miR156b-regulated SBP genes in anthocyanin biosynthesis. Because these genes are present in multiple copies in all land plants examined to date and SBP/SPL genes are also involved in several other aspects of plant biology, defining the complete scope of their function and role in shaping their activity using the reporter lines and mutants mentioned here is necessary.

## Materials and methods

### Plant materials and treatments

Seven-year-old field-grown wine grape variety ‘Cabernet Sauvignon’ (*Vitis vinifera*) vines were used as experimental materials. The treatments in this study were divided into the following groups: (i) control (total irrigation amount from 4 WAF to grape ripening: 0.52 m^3^ water per vine; leaf water potential Ψb ≥ −0.2 MPa), (ii) drought (0.31 m^3^ water per vine; −0.4 MPa ≥ Ψb ≥ −0.6 MPa), and (iii) drought + NDGA (drought treatment-combined grape berries evenly sprayed with 50 μmol/l of NDGA every 5–7 days from veraison). Thirty-five berries from each treatment group were collected at 8, 10, 12, 14, and 16 WAF. Subsequently, they were immediately frozen in liquid nitrogen and stored at −80°C until further use. All experiments were conducted in triplicate, and 30 vines were used for each repetition.

Transgenic and WT *A. thaliana* plants were grown in soil for 3 weeks under well-watered conditions, kept without water for another 10 days, and then re-watered for 2 days. The rosette leaf and petiole phenotypes were observed, and the anthocyanin content was determined.

For phenotyping the transgenic ‘Gamay’ (*V. vinifera*) grape callus during a normal growth condition or in response to simulated drought stress, WT, vv-miR156b overexpressing (MIR156b-OE), and knockdown (miR156-STTM) transgenic grape callus lines were used. WT and transgenic grape callus tissues were cultured on a Murashige and Skoog (MS) solid medium and subcultured every 3 weeks. Callus portions (~0.5 g) were maintained on MS medium for 7 days after subculturing and then transferred to MS medium or MS medium supplemented with 150 mM mannitol for 14 days. All tissues remained under a 16/8-h light/dark photoperiod (light intensity, 95 μmol m^−2^ s^−1^) at 24°C.

### Extraction of RNA and quantitative RT–PCR analysis

The isolation of total RNA and synthesis of first-strand cDNA were performed following our previous study [31]. To check gene and *MIR156b* expression, qRT–PCR analysis was performed using a QuantStudio 6 Flex Real-Time PCR System (Applied Biosystems, Life Technologies, Carlsbad, CA, USA) according to the protocol recommended by Applied Biosystems using SYBR Premix Ex Taq (Takara Bio, Beijing, China). *VvACTIN* [62] and *VvUBI* [63] served as internal controls. *AtACTIN* was used as a reference control for semi-qRT–PCR in *Arabidopsis* [64].

cDNAs were synthesized using an miR156 stem-loop primer and SuperScript III RT–PCR technology to assess the expression of mature miR156b using stem-loop qRT–PCR. As previously stated, a specific reverse transcription primer for mature miRNAs with a stem-loop structure was created [65]. qRT–PCR reactions (20 μl containing 10 ng cDNA and the vv-miR156b/stem-loop universal-R primer set) were processed using the QuantStudio 6 Flex Real-Time PCR System (Applied Biosystems, Life Technologies, Carlsbad, CA, USA), as described above. Here, 5.8S rRNA and U6 were used as the reference [66]. Each reaction was conducted in three independent biological replicates and verified using melting curve analysis. The relative expressions were calculated using the 2^−ΔΔCT^ method. Primers used are listed in Supplementary Data Table S1.

### Subcellular localization

The *VvSBP8*/*13* CDS region, minus the stop codon, was cloned from ‘Cabernet Sauvignon’ cDNA and inserted into the pCAMBIA2300 vector containing the CaMV 35S promoter and GFP protein. *p35S:VvSBP8-GFP* and *p35S:VvSBP13-GFP* plasmids were introduced into tobacco (*Nicotiana benthamiana*) leaves via *Agrobacterium*-mediated transient transformation to observe the GFP signal after incubation in 1 day of darkness and 2 days under light conditions of 16-h light/8-h dark. *p35S:GFP* and nuclear localization marker vectors containing mCherry-NLS were used as negative and positive controls, respectively. A confocal laser scanning microscope (TCS SP8-SE, Leica, Wetzlar, Germany) was used to detect GFP and mCherry signals.

### Plasmid construction, grape callus and fruit, and *Arabidopsis* transformation

To obtain the vv-miR156b-OE vector, the *pre-miR156b* (*MIR156b*) sequence, which includes ~200 bp upstream and downstream flanking regions of the miRNA, was extracted as a potential miRNA precursor. The sequence was inserted into the pCAMBIA1300 vector under the control of the *AtRD29A* promoter to generate the construct *RD29A:MIR156b*. The *VvSBP8/13* CDS region was inserted into the pCAMBIA2300 vector containing the CaMV 35S promoter to generate constructs *VvSBP8-OE* and *VvSBP13-OE*. To construct the RNAi vector, 430-bp sense and 430-bp antisense VvSBP8 fragments, as well as 435-bp sense and 435-bp antisense VvSBP13 fragments, were amplified and subcloned into the RNAi empty vector for transformation. To investigate transgenic miRNAs in grape calli, the *MIR156b* sequence was inserted into the pCAMBIA2300 vector containing the CaMV 35S promoter to obtain miR156b-OE. The function of vv-miR156b was blocked using STTM technology to obtain miR156b-STTM calli (Supplementary Data Fig. S1). The structure of miR156b-STTM was designed according to the procedure described by Yan *et al*. [67]. miR156b-STTM was inserted into BamHI–SalI-cut pCAMBIA1300 with a 2x35S promoter to generate the recombinant plasmid miR156b-STTM.

These recombinant plasmids were transformed into *Agrobacterium* strain GV3101 and then used to transform *A. thaliana* using the floral dip method [68]. *T*_3_ homozygous lines were used for further studies.

The plasmids *MIR156b-OE*, miR156b-STTM, *VvSBP8-OE*, *VvSBP13-OE*, *VvSBP8-CRISPR*, *VvSBP13-CRISPR*, and EV were introduced into *Agrobacterium tumefaciens* strain EHA105 and then transformed into grape calli and berries. The stable transformation of grape calli was performed as follows: grape calli were incubated in an *Agrobacterium* suspension under low-speed shaking for 10 min, filtered through a cell sieve, dried and blotted on sterile filter paper, and then transferred to a cured 1/2 GS medium containing 200 μM acetosyringone. Grape calli were transferred to GS medium with plant-carrier resistance to 60 mg/l kanamycin and 200 mg/l cefotaxime after 3 days of co-culture in the dark. Five weeks later, most calli began to necrotize and die. The fraction of grape calli proliferating on the screening medium was subcultured on fresh GS medium containing plant-carrier antibiotics with 60 mg/l kanamycin and 200 mg/l cefotaxime. Calli cells that had differentiated from various places (or from various dishes) in the culture media were transferred to various lines and subcultured at intervals of 15 days using kanamycin screening. Resistant calli with consistent growth after five serial subcultures were subjected to transgenic PCR detection and qRT–PCR investigation for gene expression and stem-loop qRT–PCR for the detection of mature miR156b. Transient transgenic grape berries were obtained using *Agrobacterium*-mediated transformation according to the method described by Dai *et al*. [69]. Detached pre-veraison grape berries with consistent development and pedicels (~2 mm) were collected and cleaned. Their pedicels (~2 mm) were placed into an *Agrobacterium* solution to incubate for 7 days at 25 ± 0.5°C, with light period 16 h/8 h day/night and light ~50 μmol m^−2^ s^−1^. Primers used in this study are listed in Supplementary Data Table S1.

### Determination of anthocyanin compounds

The total anthocyanin content in grapevine berry skin was determined following the method described by Guo *et al*. [31]. The anthocyanin contents in grape calli and *Arabidopsis* were determined using the following method: 0.1 g fresh calli or leaves was mixed with 200 μl extraction solution (18% *n*-propyl alcohol, 1% hydrochloric acid, and 81% deionized water) and extracted at 95°C for 2 min, followed by resting for 5 min in an ice bath and oscillation at 4°C overnight. After centrifugation at 5000 rpm for 10 min, the absorbance (A) of each supernatant was measured at 535 and 650 nm and calculated using the following equation:

A = A_535 nm_ − A_650__nm_.

Anthocyanins content = A/ε × *V*/*M* × 106 (ε = 4.62 × 10^4^).

Where *V* and *M* represent the volume of the extraction solution and the fresh weight of the sample, respectively.

### Yeast one-hybrid assay

The miR156b promoter fragment was synthesized by Sangon Biotech (Shanghai, China) and contained three repeats of ABRE elements, which were ligated into the pAbAi vector. The full-length CDS of the *VvAREB2* region without a stop codon was cloned and ligated into the pGADT7 vector. All primers used for the Y1H assays are listed in Supplementary Data Table S1. The assay was conducted using the Matchmaker™ Gold Yeast One-Hybrid Library Screening System (OeBiotech). The linearized pBait-AbAi plasmid was transformed into the yeast strain Y1H Gold and screened on SD/−Ura selective medium. The optimal concentration of the yeast cell growth inhibitor aureobasidin A was used as a screening marker.

### Transient dual-LUC reporter assay

To evaluate the interaction between VvAREB2 and the *vv-MIR156* promoter *in vivo*, the *vv-MIR156b* promoter and a mutated version of the *vv-MIR156* promoter were inserted into the BamHI/NcoI sites of the pGreenII 0800-LUC vector at the 5′ end of the *LUC* gene. The *VvAREB2* CDS without a stop codon was inserted into the pCAMBIA2300 vector. As the reference control, the pGreenII 0800-LUC vector contained the *REN* gene under the control of the 35S promoter.

To evaluate the suppression of the *VvSBP8/13* target genes by miR156b, the *vv-MIR156b* fragment was inserted into pCAMBIA2300 under 35S. Subsequently, each *VvSBP8* and *VvSBP13* ORF sequence driven by 35S was connected and inserted into the BamHI/NcoI sites of the pGreenII 0800-LUC vector at the 5′ end of the *LUC* gene. As a reference control, the pGreenII 0800-LUC vector contained the *REN* gene under the control of the 35S promoter.

To verify the *VvSBP8/13* cleavage sites of miR156b, potential *VvSBP8/13* cleavage sites were inserted into the AvrII/AgeI sites of the pGreen-dualluc-ORF-sensor as described by Liu *et al*. [70]. A negative control (SNC) with disruption of the *VvSBP8/13* cleavage site while maintaining an identical amino acid sequence was inserted into the AvrII/AgeI sites of the pGreen-dualluc-ORF-sensor vector. The vv-MIR156 overexpression construct was inserted into the pCAMBIAM2300 vector containing the CaMV 35S promoter. The pGreenII 0800-LUC and pGreen-dualluc-ORF-sensor vectors carried the *Renilla* luciferase (*REN*) gene under the control of the CaMV 35S promoter as a positive control. Primers used are listed in Supplementary Data Table S1. All constructs were generated using 4-week-old tobacco leaves by the mediated transient transformation of the *A. tumefaciens* strain GV3101. LUC and REN activities were measured using a dual-luciferase reporter gene assay kit (Yeasen).

### Split-LUC assay

In the split-LUC analysis, the CDS regions encoding *VvMYC1* and *VvMYBA1* were amplified and cloned into pCAMBIAM1300-nLUC and pCAMBIAM1300-cLUC vectors, respectively. For the split-LUC experimental analysis of the interaction of VvMYC1 with VvMYBA1, *35S:VvMYC1-nLUC*, and *35S:cLUC-VvMYBA1* were co-expressed in tobacco leaves. Split-LUC assays were used to determine the effect of VvSBP8/13 on the binding of VvMYC1 and VvMYBA1. 35S:VvSBP8/13-YFP or 35S:YFP constructs were co-expressed in the presence of *35S:VvMYC1-nLUC* and *35S:cLUC-VvMYBA1*. *Pro35S:P19* was co-infiltrated to inhibit gene silencing. Cultures were co-injected into 4-week-old tobacco leaves. LUC and REN activities were measured using a dual-LUC reporter gene assay kit (Yeasen).

### Bio-layer interferometry assay

The binding affinity between VvAREB2 and *vv-MIR156b* promoter was measured using a BLI assay with Octet 96 (ForteBio, Silicon Valley, CA, USA). VvAREB2 (2 μg/ml) was immobilized to capture biosensors and balanced with PBST (10 mM PBS + 0.02% TW-20, pH 7.4). Biosensors were sequentially exposed to the element probe and its mutated probe. Different probe concentrations (15.625, 31.25, 62.5, 125, 250, and 500 nM) were used to quantify protein interactions, followed by washing with PBS, 5 M NaCl, and PBST. The binding affinity (*K*_D_) in this experiment was determined using custom software written in Blitz System software (ForteBio).

### Yeast two-hybrid assay

The CDS of *VvSBP8/13* were cloned into the pGBKT7 and pGADT7 vectors, and those of *VvMYC1* and *VvMYBA1* were cloned into the pGBKT7 and pGADT7 vectors, respectively. The resulting BD-VvSBP8/13 and AD-VvMYBA1 and BD-VvMYC1 and AD-VvSBP8/13 recombinant plasmids were co-transformed into the yeast strain AH109 (Clontech). Yeast growth status was examined using SD/−Trp−Leu−His−Ade medium.

### Yeast three-hybrid assay

The CDS of *VvMYBA1* was cloned into a pGADT7 vector. The pBridge vector was used as the BD vector to express VvMYC1 and VvSBP8/13. VvMYC1 is expressed in yeast host cells driven by the constitutive promoter ADH1, while VvSBP8/13 is expressed under the control of the *Met25* promoter. The yeast strain AH109 was transformed and screened for co-transformed colonies on SD/−Trp−Leu plates. Yeast cells were grown on SD/−Trp/−Leu/−His/−Ade plus 1 mM Met and selective SD/−Trp/−Leu/−His/−Ade/−Met media.

### Statistical analysis

All differences in data were statistically evaluated using one-way analysis of variance (ANOVA), Duncan’s multiple range test (DMRT), and Tukey’s test (*t*-test) using SPSS 17.0. Data are expressed as mean ± standard deviation.

## Acknowledgements

This work was supported by the National Natural Science Foundation of China (grants 32371924, 32260727, and 31801811), the Key Research and Development Program of Shaanxi Province (grant 2023-GHZD-17), the Key Research and Development Programme of Xinjiang Province (grant 2022B02034-3), and the China Agriculture Research System (grant CARS-29-zp-6). We are grateful to Dr Jing Zhang, Dr Yangyang Yuan, Miss Haiying Wang, and Miss Beibei He (Horticulture Science Research Center, Northwest A&F University, Yangling, China) for providing professional technical assistance; We also thank Prof. Yaoguang Liu (South China Agricultural University), Prof. Mingzhi Yang (Yunnan University), Prof. Dongqing Xu (Nanjing Agricultural University), and Prof. Chao Ma (China Agricultural University) for gifting us materials and plasmids.

## Author contributions

T.F.X., J.F.M., and Z.W.Z. conceived and designed the experiments. S.H.G., M.Z., M.X.F., G.P.L., X.Q.T., and R.H.R. performed the experiments, and S.H.G. and M.Z. analyzed the data. S.H.G., T.F.X., M.Z., L.T., Y.L.F., and J.F.M. wrote and edited the manuscript. All authors reviewed and approved the manuscript.

## Data availability

All relevant data generated or analyzed are included in the manuscript and the supporting materials. The sequence information used in this study can be obtained from the NCBI database (https://www.ncbi.nlm.nih.gov/). The GenBank accession numbers are as follows: *VvAREB2* (XM_010655778.2), *VvUFGT* (XM_002276999.4), *VvLDOX* (NM_001281218.1), *VvDFR* (XM_002281822), *VvSBP8* (XM_002278476.1), *VvSBP13* (XM_002274466.1), *VvACTIN *(XM_002282480.4), *VvUBI* (XM_002273532.2), and *AtACTIN* (AT3G18780).

## Conflict of interest

None declared.

## Supplementary data

Supplementary data is available at *Horticulture Research* online.

## Supplementary Material

Web_Material_uhad293
