# Supplementary material for: miR156b-targeted VvSBP8/13 functions downstream of the abscisic acid signal to regulate anthocyanins biosynthesis in grapevine fruit under drought
Source: Hortic Res. 2024 Jan 2;11(2):uhad293. doi: 10.1093/hr/uhad293 (PMC10873574; doi:10.1093/hr/uhad293)
Supplement: Web_Material_uhad293 [file web_material_uhad293.zip › Supporting__information_DATA_final_2023.7.12-2.docx]

**Supplementary information**

**Figure S1.** Diagrams of vv-miR156b-STTM with spacer lengths.

**Figure S2.** The anthocyanins content and miR156b expression upon drought stress in *vv-MIR156b* transgenic *Arabidopsis* lines.

**Figure S3.** Visual analysis of cis-elements of vv-miR156s promoter.

**Figure S4.** Association and dissociation curves of VvAREB2 with *vv-MIR156b* mutant promoter by BLI assay.

**Figure S5.** Expression change of other *VvSBPs* member under drought stress.

**Figure S6.** Detection of protein in *VvSBP8* and *VvSBP13* overexpressing grape calli.

**Figure S7.** Anthocyanin accumulation analysis of *VvSBP8/13 Arabidopsis* overexpressors.

**Figure S8.** Mature vv-miR156 transcript abundance determined by stem-loop qRT-PCR in both VvSBP8-OE+MIR156b-OE and VvSBP13-OE+MIR156b-OE transgenic calli.

**Table S1.** List of primers used in this study.

**Figure S1.**


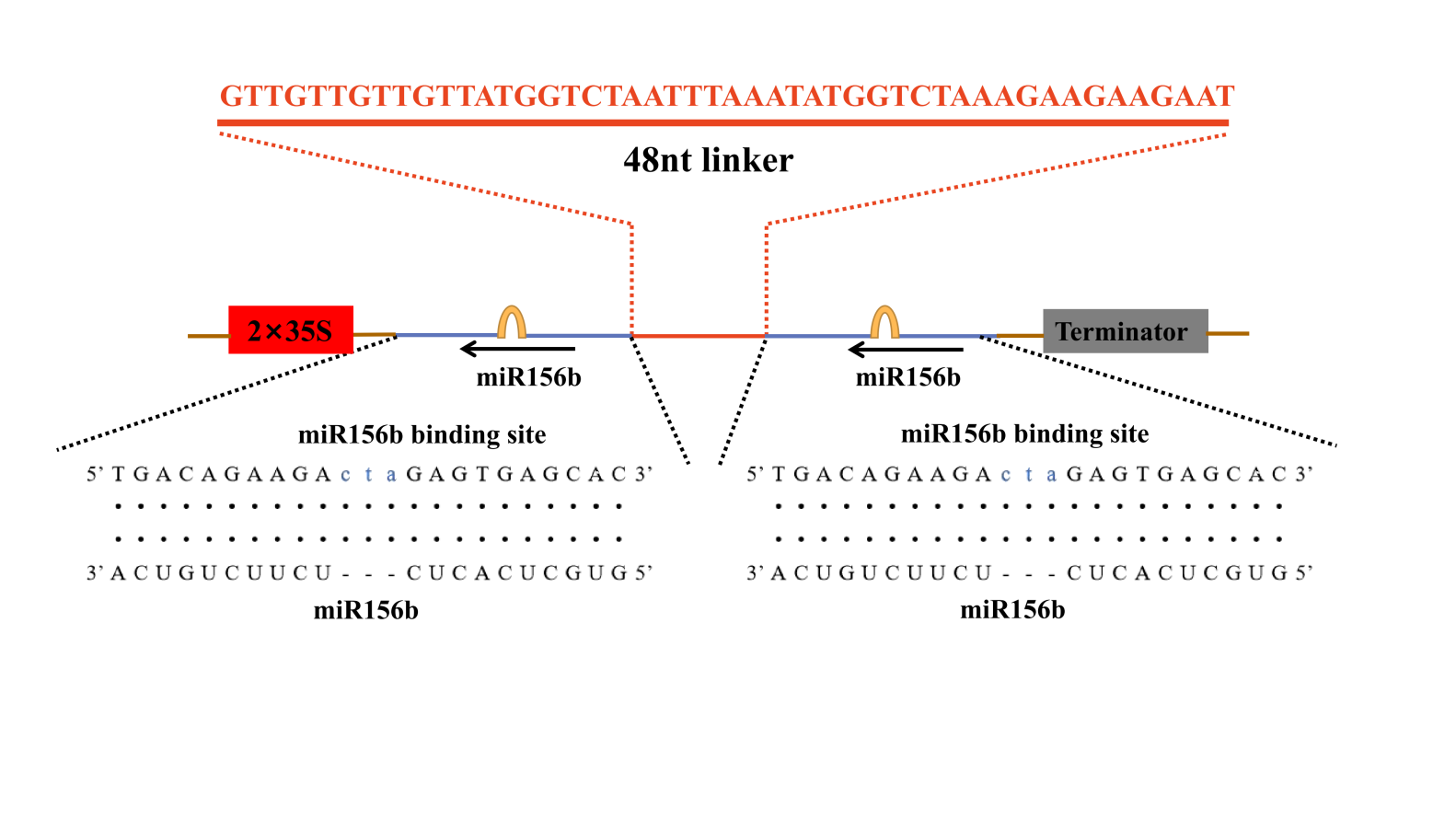


**Figure S1.** Diagrams of the vv-miR156b-STTM with spacer lengths. Orange indicates 48nt spacer sequence. The enhanced double 35S promoter was used for expressing STTM in grape.

**Figure S2.**


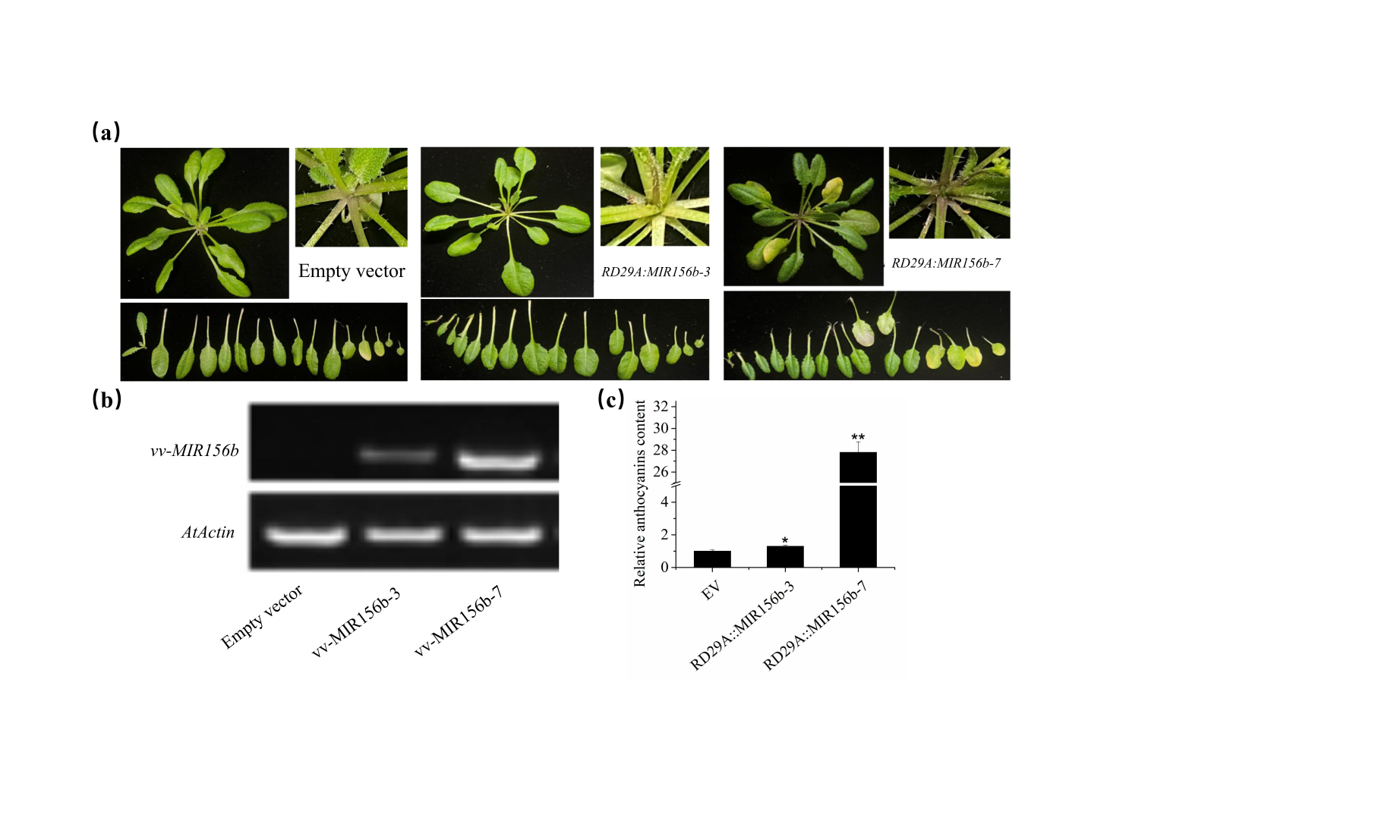


**Figure S2.** The anthocyanins content and miR156b expression upon drought in *vv-MIR156b* transgenic *Arabidopsis* lines. (a) Morphological characterization and fully-expanded rosette leaves of *vv-MIR156b* transgenic and EV lines upon drought. EV: Empty vector; *RD29A:MIR156b*: vv-miR156b precursor driven by *Arabidopsis AtRD29A* promoter. (b) PCR amplification of *vv-MIR156b* fragment by semi-qRT-PCR. *AtActin* was used as reference. (c) The relative anthocyanins content drought-treatment EV and transgenic lines. Error bars represent standard deviation of three biological replicates. Statistically significant differences were determined by t-tests: *P<0.05; **P<0.01.

**Figure S3.**


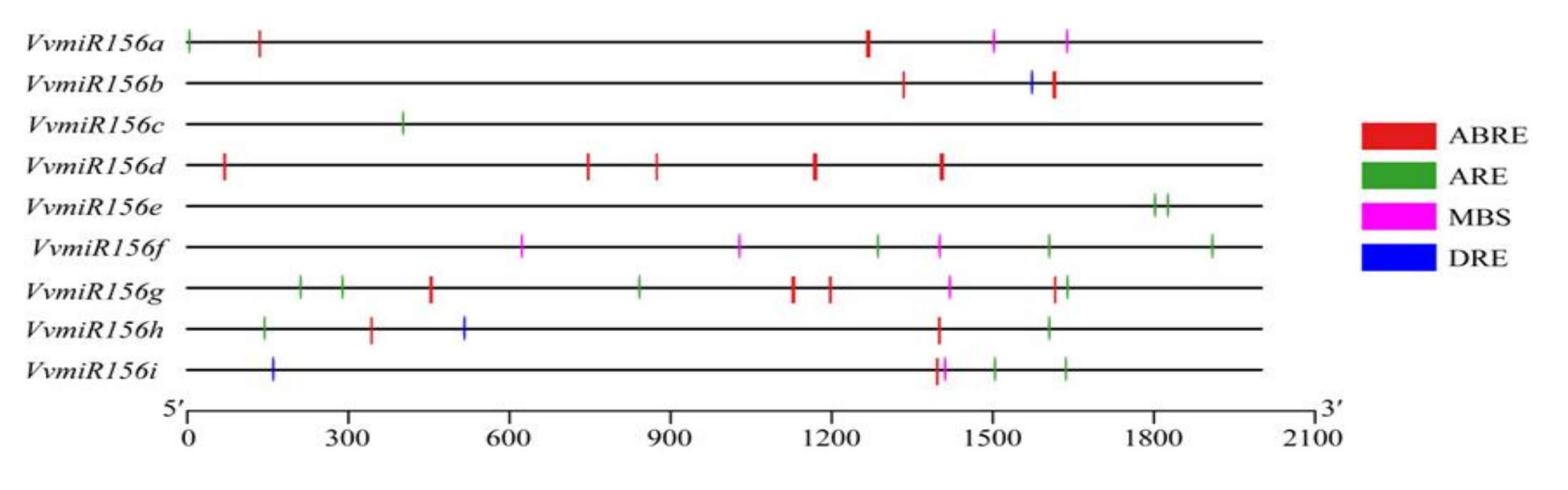


**Figure S3.** Visual analysis of cis-elements of vv-miR156s promoter. The 2000 bp DNA fragments upstream of the miR156s are analyzed using the online analysis software PlantCARE. Different cis-acting elements are displayed. The different colored markers indicate different predicted cis-acting elements.

**Figure S4.**


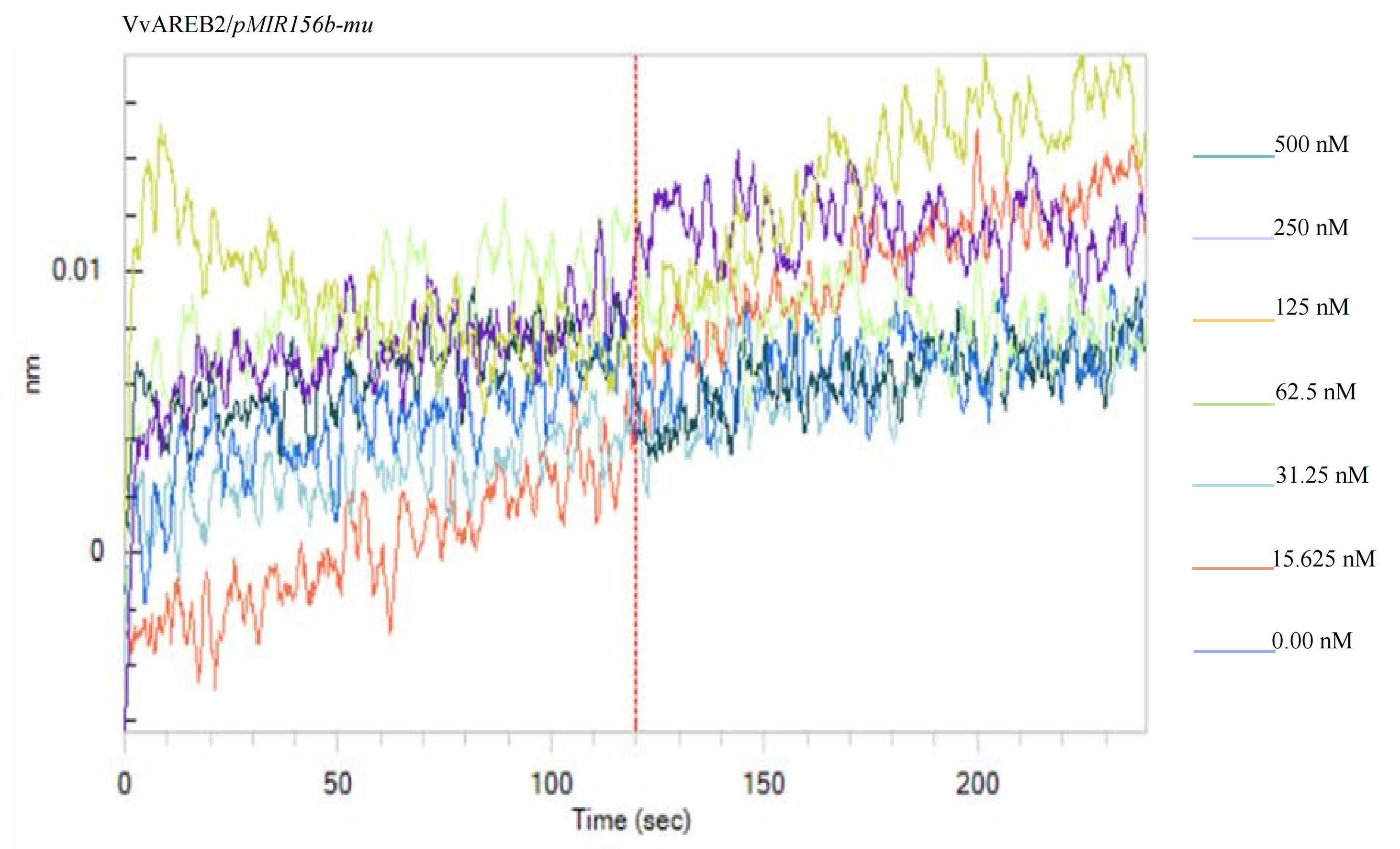


**Figure S4.** Association and dissociation curves of VvAREB2 with *vv-MIR156b* mutant promoter by BLI assay. Biotinylated *MIR156b* promoter mutant was immobilized on streptavidin (SA) biosensor tips and incubated over a range of concentrations (15.625–500 nM) of soluble VvAREB2 protein.

**Figure S5.** Expression change of other *VvSBPs* member under drought.

**







**

**







**

**







**

**







**

**Figure S6.**


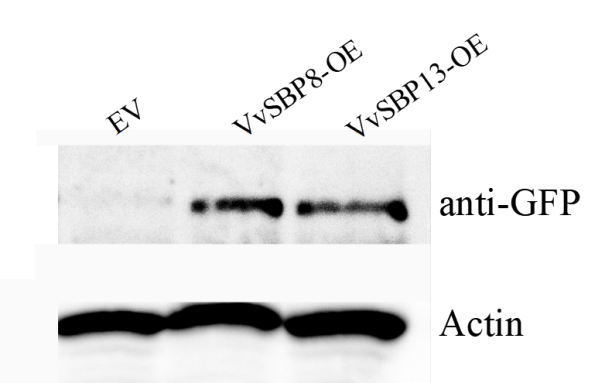


**Figure S6.** Detection of protein in overexpressing *VvSBP8* and *VvSBP13* grape calli. Western blot analysis of protein levels of VvSBP8 or VvSBP13 individual transgenic grape calli lines in figure 5c. Total protein analyzed by SDS-PAGE and immunoblotting with α-GFP and α-ACTIN antibodies. ACTIN was detected as loading control.

**Figure S7.**


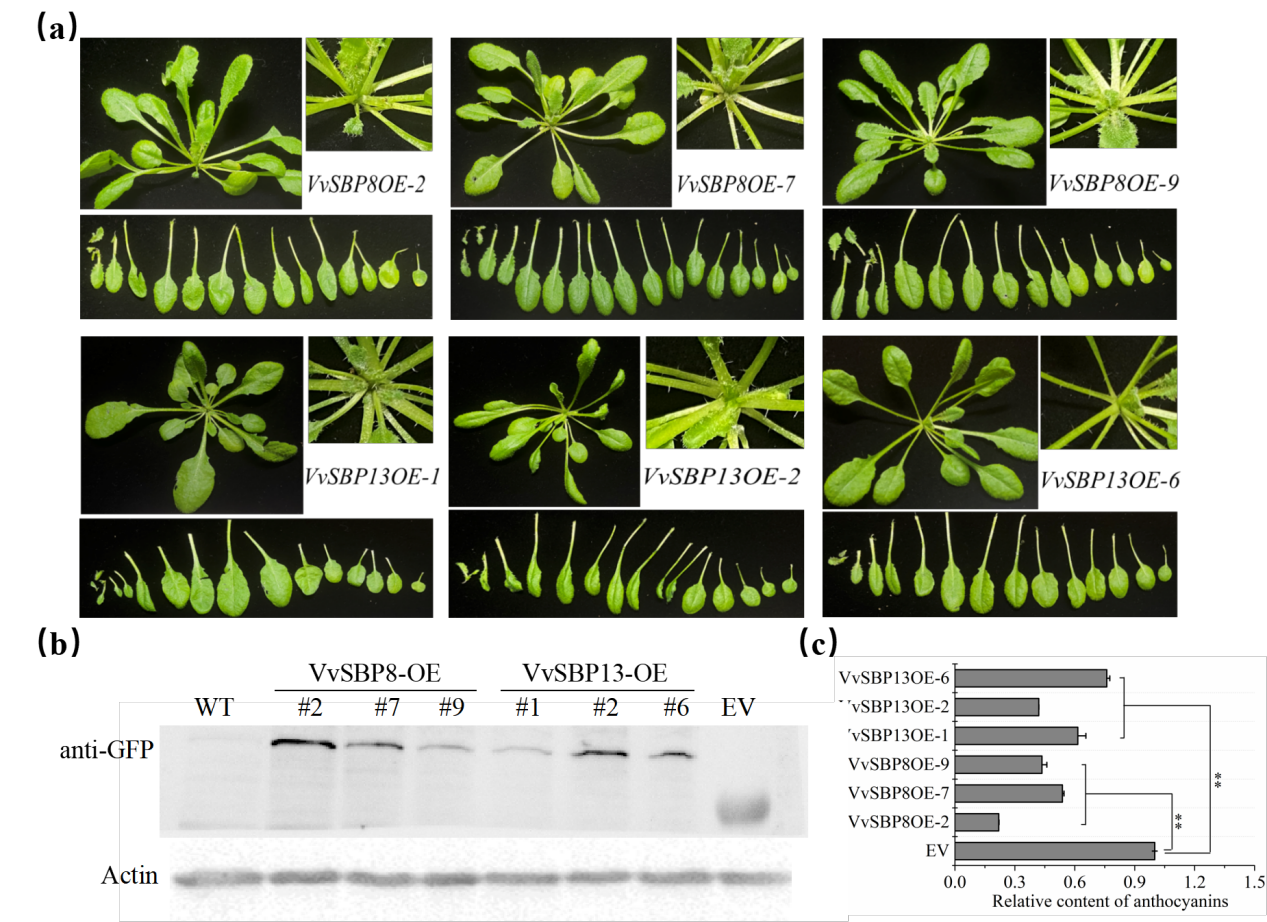


**Figure S7.** Anthocyanin accumulation analysis of VvSBP8/13 *Arabidopsis* overexpressors. (a) The color and rosette leaves of *VvSBP8/13* overexpessors. (b) Western blot analysis of protein levels. Total protein was analyzed by SDS-PAGE and immunoblotting with α-GFP and α-ACTIN antibodies. ACTIN was detected as loading control. (c) Relative anthocyanins content was calculated in overexpressors and empty line (EV) that was used as the control with the relative content level set as 1.0. Error bars represent standard deviation of three biological replicates. Statistically significant differences were determined by t-tests: **P<0.05*; ***P<0.01*.

**Figure S8.**


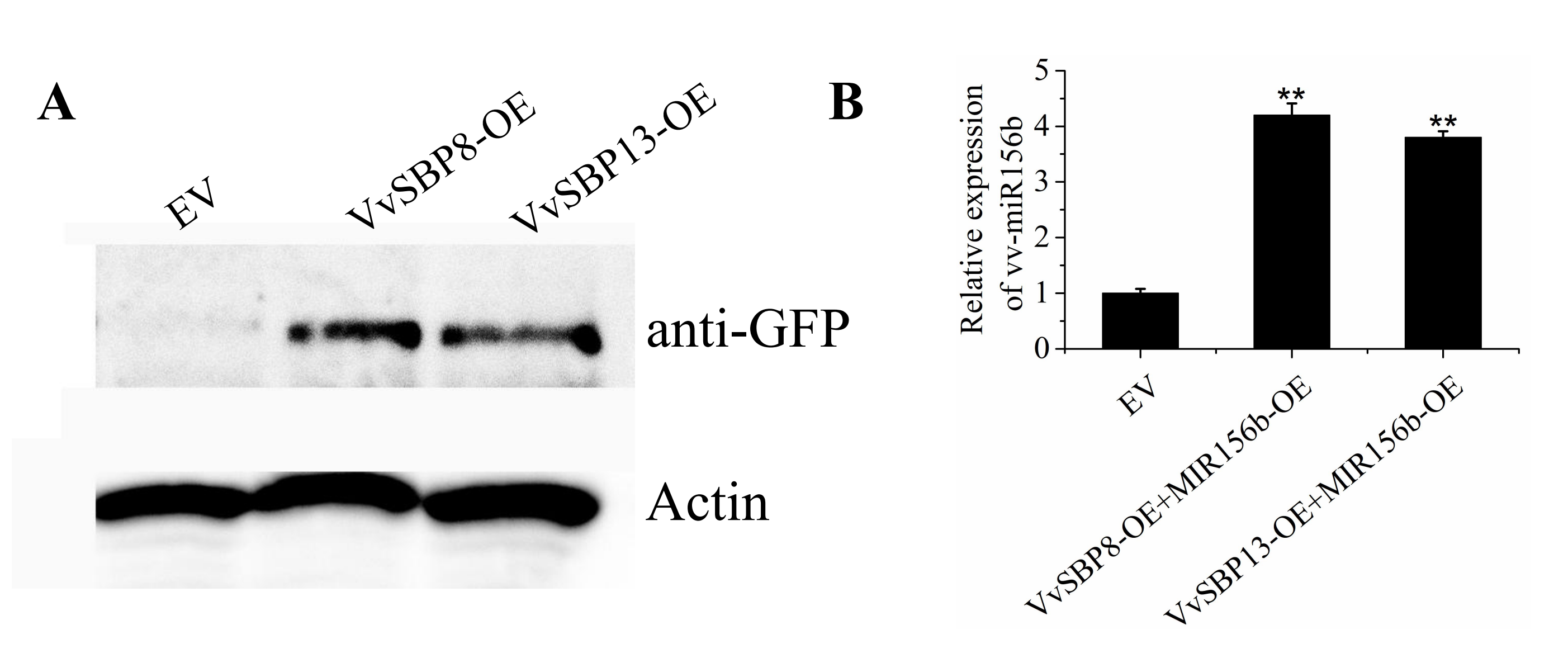


**Figure S8.** Detection of vv-miR156 transcript abundance in both VvSBP8-OE+MIR156b-OE and VvSBP13-OE+MIR156b-OE transgenic calli. The expression analysis of mature vv-miR156b in VvSBP8+vv-miR156b or VvSBP13+vv-miR156b co-transformed grape calli compared with EV calli line detected by stem-loop qRT-PCR. 5.8S rRNA and U6 were used as the internal control. EV: empty vector. Error bars represent biological triplicates (±SD).
